# Benchmarks for flexible and rigid transcription factor-DNA docking

**DOI:** 10.1186/1472-6807-11-45

**Published:** 2011-11-01

**Authors:** RyangGuk Kim, Rosario I Corona, Bo Hong, Jun-tao Guo

**Affiliations:** 1 Department of Bioinformatics and Genomics, College of Computing and Informatics, University of North Carolina at Charlotte, Charlotte, North Carolina, USA; 2 School of Electrical and Computer Engineering, Georgia Institute of Technology, Atlanta, Georgia, USA

## Abstract

**Background:**

Structural insight from transcription factor-DNA (TF-DNA) complexes is of paramount importance to our understanding of the affinity and specificity of TF-DNA interaction, and to the development of structure-based prediction of TF binding sites. Yet the majority of the TF-DNA complexes remain unsolved despite the considerable experimental efforts being made. Computational docking represents a promising alternative to bridge the gap. To facilitate the study of TF-DNA docking, carefully designed benchmarks are needed for performance evaluation and identification of the strengths and weaknesses of docking algorithms.

**Results:**

We constructed two benchmarks for flexible and rigid TF-DNA docking respectively using a unified non-redundant set of 38 test cases. The test cases encompass diverse fold families and are classified into easy and hard groups with respect to the degrees of difficulty in TF-DNA docking. The major parameters used to classify expected docking difficulty in flexible docking are the conformational differences between bound and unbound TFs and the interaction strength between TFs and DNA. For rigid docking in which the starting structure is a bound TF conformation, only interaction strength is considered.

**Conclusions:**

We believe these benchmarks are important for the development of better interaction potentials and TF-DNA docking algorithms, which bears important implications to structure-based prediction of transcription factor binding sites and drug design.

## Background

Transcription factors (TFs) play key roles in the regulation of gene expression through binding to specific DNA sequences known as transcription factor binding sites (TFBSs) [1-3]. At the genomic level, the interactions between TFs and their binding sites in target genes (TGs) form multi-layered regulatory networks, in which TFs and TGs are represented as nodes and direct links between TFs and TGs correspond to regulatory interactions [4-7]. Although these transcriptional networks can be studied with one or more particular focuses, such as the structure, function, and/or evolution, the fundamental step in network construction is the identification of transcription factor binding sites. Computational identification of TFBSs on a genomic scale has been considered as a promising strategy for delineating these networks and remains one of the primary challenges in post-genomic bioinformatics [8,9]. Most of the current computational methodologies for TFBSs prediction are sequence-based; however structure-based TFBS prediction is gaining popularity [10-17]. Currently, structure-based approaches rely on resolved TF-DNA complex structures. Despite rapid technological advances in experimental structure determination, the number of experimentally solved TF-DNA complex structures remains scant in Protein Data Bank (PDB)[18]. Computational docking represents a useful tool for studying the mechanisms of molecular recognition in complex structures. Previous studies have demonstrated that molecular docking can obtain accurate complex structures for protein-protein, protein-peptide, and protein-ligand interactions [19-22]. However, protein-DNA docking, especially TF-DNA docking, still represents a largely unexplored vista when compared to the progress made in protein-protein and protein-ligand docking [13,23-25].

In structural bioinformatics, benchmarks are routinely used for assessing systematic performance of predictive approaches such as fold recognition [26,27], protein-ligand docking [28], and protein-protein docking [29,30]. Carefully designed benchmarks with a wide variety of test cases can provide objective evaluation, help identify the strengths or weaknesses of different methods, and facilitate the development of better algorithms and parameter optimization [31]. Recently, a general protein-DNA docking benchmark consisting of 47 protein-DNA test cases has been developed [32]. While this benchmark contains well-defined test cases for evaluating protein-DNA docking in general, the unique characteristics of transcription factors and the imperative goal of structure-based TF-binding site prediction call for a TF-specific docking benchmark. Transcription factors represent one of the largest groups of proteins in most genomes and form a distinct group of DNA-binding proteins in terms of sequence specificity and flexibility [4,5,33]. It is well known that DNA-binding proteins encompass diverse functional categories [34-36] including enzymes involved in DNA replication, recombination, cleavage, repair and other nucleic acid metabolizing processes. Some of these enzymes are sequence-independent when binding to DNA molecules as in the cases of polymerases, DNase I, and histone binding proteins, while others are more stringent sequence-specific enzymes, such as HhaI methyltransferases and most of the type II restriction endonucleases [34,35,37,38]. Transcription factors, on the other hand, recognize specific binding sites while allowing certain degrees of variations.

Moreover, different interaction or binding "modes" have been reported for transcription factors, restriction endonucleases (REs), and non-specific (NS) DNA binding proteins [36,39,40]. In a recent study, Contreras-Moreira et al. showed that restriction endonucleases have a "substantially larger proportion of indirectly readout bases" when compared with other transcription factor superfamilies [40]. In the general protein-DNA docking benchmark by van Dijk and Bonvin, most of the restriction endonucleases are classified into the difficult category and half of the 'Difficult' targets are restriction endonucleases due to their large conformational differences between bound and unbound protein structures, suggesting restriction endonucleases have different binding mechanisms to a certain degree [32]. Our analysis of residue-base interactions and protein-DNA interaction interface of three major types of DNA binding proteins, TF, RE, and NS, also confirms these differences (see Methods).

To facilitate the study of the TF-DNA docking problem and structural-based TF binding site prediction, we construct two benchmarks, one for flexible TF-DNA docking using unbound TFs as the starting structures, and the other one for rigid docking using bound TF conformations as the starting structures, with intended applications in assessing the capability of docking programs to deal with conformational changes, and evaluating docking algorithms and energy potentials [13]. Both benchmarks are constructed from a unified set of 38 TF-DNA complexes and corresponding unbound TF structures.

**Figure 1:**
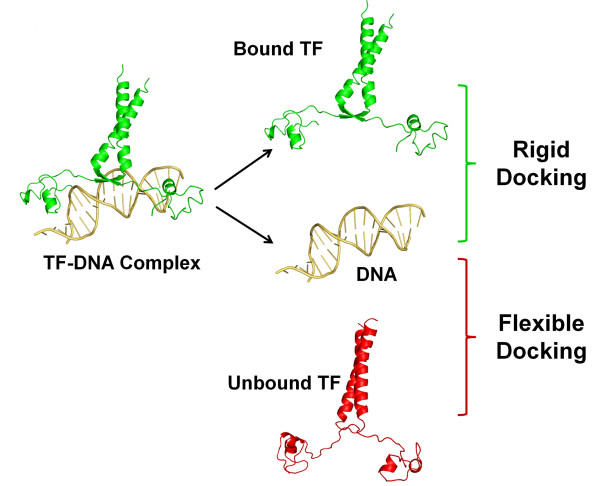
**Schematics for construction of rigid and flexible TF-DNA docking test cases**. The flexible docking test cases are constructed using unbound TF structures and bound DNA structures while the rigid docking test cases use both bound TF and bound DNA conformations.

Besides specific interactions between protein residues and DNA bases, it has been well accepted that DNA deformations/shapes or 'indirect readout' play important roles in protein-DNA interaction [40-43]. In our benchmarks, we use the bound DNA structures instead of the canonical B-form DNA structures for benchmark construction (Figure 1). We have demonstrated previously in our semi-flexible protein-DNA docking that near-native DNA structures can be modeled from representative DNA conformations compiled from known DNA structures in protein-DNA complexes [13]. Therefore, the contribution of indirect readout in TF-DNA interaction is not considered as a variable in grouping the test cases. For both flexible and rigid docking test cases, we consider the strength of TF-DNA interactions in assigning levels of difficulty. If the interaction interface is small, the probability of a correct prediction is also low. For flexible cases, the conformational difference between the unbound and bound TF structures serves as an additional factor for accessing the degrees of docking difficulty as larger structural differences between bound and unbound forms require more efficient handling of conformational changes in docking prediction.

To our knowledge, our benchmarks are the first large sets with test cases compiled *ad hoc *for TF-DNA complexes. The cases represent a diverse group of transcription factors (15 SCOP superfamilies) [44]. These two benchmarks are different from the general protein-DNA docking benchmark by Van Dijk and Bonvin in that besides transcription factors, their benchmark also consists of restriction endonucleases and other types of DNA modification enzymes [32]. Though a set of TF-DNA complexes was used to perform docking studies by Aloy *et al*., the set is small (8 cases) and is restricted to repressors [45]. Another unique feature of our benchmarks lies in that this carefully selected, unified set of test cases can be used for both rigid docking and flexible docking. We believe that our benchmarks will serve as a test ground for TF-DNA docking studies, which has important implications in structure-based TF binding site prediction. The test cases in PDB format are available for download at http://bioinfozen.uncc.edu/tf-dna-benchmarks.

## Methods

### TF-DNA complex structures and TF-DNA binding units

The first step in test case selection is to cull sequence-specific TF-DNA complex structures from PDB [18]. Since the classification of some DNA-binding proteins in PDB is sometimes ambiguous, for example, transcription factors *Escherichia coli *SigmaE Region 4 (2H27) and the ribbon-helix-helix domain of *Escherichia coli *PutA (2RBF) are classified as "transferase" and "oxidoreductase" respectively in PDB, we combined information from PDB keywords, UniProt [46] keywords, and Gene Ontology (GO)[47] terms with manual inspection to identify all TF-DNA complexes in PDB.

Each test case in our benchmarks is a TF-DNA binding unit. A TF-DNA binding unit is defined as an entity of a DNA double helix and one or more TF-chains that interact with each other with at least three residue-residue contacts based on a heavy atom distance cutoff of 4.5 Å. If a PDB entry has two or more TF-DNA binding units, a representative TF-DNA binding unit is carefully selected based on the detailed protein-DNA interaction, visual inspection and literature search. For example, 3HDD (engrailed homeodomain-DNA complex) has two TF-DNA binding units. One is in the middle of the DNA helix while the other one binds to the edge of the DNA structure (Additional file 1, Figure S1). The one close to the middle of the DNA has more protein-DNA interactions and is selected as a test case. For presentation purpose, the TF chain or chains in a TF-DNA binding units are dubbed as a TF unit in our study.

### Structure comparison and TF-DNA interaction interface

Structure alignment is carried out with TM-align [48]. TM-align algorithm uses TM-score instead of the commonly used RMSD (Root Mean Square Deviation) for alignment optimization. TM-score is more sensitive to global structure topology than to local structure changes [48,49]. The RMSD between two TF chains (RMSD_c_) or two TF units (RMSD_u_) is calculated with the alpha carbons of the amino acids that are aligned by the global sequence alignment program NEEDLE in EMBOSS package [50].

The TF-DNA interface or the buried surface area (BSA) of a TF-DNA binding unit is determined by calculating the difference in solvent accessible surface area (ASA) between separate TF and DNA structures and TF-DNA complexes, *i.e*.

$$\text{BSA}=0.5\times \left(\text{AS}{\text{A}}_{\text{TF}}+\text{AS}{\text{A}}_{\text{DNA}}-\text{AS}{\text{A}}_{\text{TF}-\text{DNA}}\right).$$

The solvent accessible surface areas are measured with POPS using default parameters [51]. The number of residue-base contacts (NRBCs) is defined as the number of residues that are in contact with a DNA base through sidechains with a heavy atom-heavy atom distance cutoff of 4.5 Å.

To investigate the interaction characteristics among different types of DNA binding proteins, we compiled three non-redundant datasets: TF, RE, and NS for transcription factors, type II restriction endonucleases, and non-specific DNA binding proteins respectively. All the complex structures are solved by X-ray crystallography method with resolutions of 3Å or better. The annotation of each complex to one of the three groups is based on the classifications in PDB [18] and literature search. The redundant entries in each set are removed using PISCES with a sequence identity cutoff of 30% [52]. The protein chains in each set (RE: 24, TF: 84, NS: 43) are shown in Additional file 2, Table S1.

**Figure 2:**
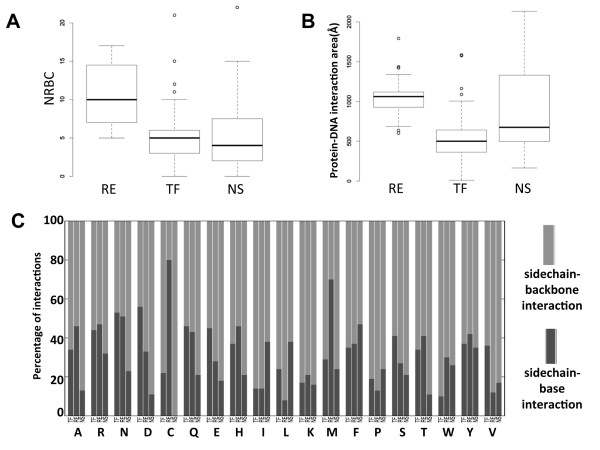
**Distributions of protein-DNA interactions in different types of DNA-binding proteins**. A: distribution of NRBC in RE, TF, and NS datasets; B: distribution of protein-DNA interaction surface in RE, TF, and NS datasets; C: percentage of residue interactions with base or backbone-only of DNA, one-letter codes are used for amino acids. RE: restriction endonuclease, TF: transcription factor, NS: non-specific.

We compared the distributions of NRBC and protein-DNA contact area among RE, TF, and NS groups. Figure 2 shows that restriction endonucleases have more residue-base contacts (Figure 2A) and larger protein-DNA interfaces (Figure 2B) than those in the transcription factor group. While the median value of the NS interface distribution falls between the median values of TF and RE (Figure 2B), the median of NRBC distribution in NS is the lowest among the three groups (Figure 2A), suggesting small ratio of base/backbone contacts with proteins in the NS group. Figure 2C shows the percentage of interactions of each residue except for glycine (no sidechain contact) with base or backbone-only in three datasets. Not surprisingly, NS has significantly lower base contacts than RE and TF groups. Large differences are also observed in about half of the residues types, alanine (A), aspartate (D), cysteine (C), glutamate (E), leucine (L), methionine (M), serine (S), tryptophan (W) and valine (V) between RE and TF protein groups (Figure 2C). These data provide further justification to the construction of TF-specific docking benchmarks.

### Selection of test cases for TF-DNA docking benchmarks

The selection process for the test cases of our benchmarks is shown in Additional file 1, Figure S2. The protein-DNA complex structures determined by X-ray crystallography with resolutions of 3.0 Å or better were first selected from PDB [18]. The protein-DNA complexes that do not have double-stranded DNA structures were identified with our previously developed program PDA (Protein-DNA Complex Structure Analyzer) [37] and removed from the set. The TF-DNA binding units were then identified as described previously. The unbound TF structures that have a sequence identity of at least 95% and coverage of 95% or better compared to the bound TFs were identified using BLAST [53]. The TF-DNA units and their corresponding unbound TF structures were clustered into different groups with a protein sequence identity cutoff of 35% using PISCES [52]. The representative test case in each group was selected based on structural qualities (high resolution, fewer missing residues/atoms in TF-DNA interface) and its *nativeness *(*e.g*. wildtype is preferred over mutants). For unbound structures, an NMR structure is chosen only if no X-ray structure is available.

Thirty-seven test cases were initially generated, which include two modeled unbound TF unit structures (1RXR and 1R69) for TF-DNA units in 1BY4 and 2OR1. The TF unit structures of 1BY4 and 2OR1 are homodimers. However, the only available unbound structures for both 1BY4 and 2OR1 are monomers. Since the conformational differences between the bound and unbound chain structure in both cases are small and for the purpose of increasing the dataset size, we modeled their unbound TF-unit structures based on their bound unit structures and unbound TF-chain structures. We also added 1AYY (ZIF 268 zinc finger), a popular test case in many studies largely due to the extensive experimental data [10-13,15,54-56], to the test cases. However, it does not have a reasonable unbound structure in PDB at this point. Thus we omit it from the flexible TF-DNA docking benchmark but will include it as soon as its unbound structure becomes available.

One flexible and one rigid TF-DNA docking benchmark were constructed using the unified 38 test cases (Figure 1, Tables 1 and 2). Because of the relatively small size of the set, we grouped the test cases into two difficulty levels (easy and hard) in both benchmarks but with different criteria and plan to expand it to three levels (easy, medium, and hard) when we have more test cases in the future. In flexible docking, conformational difference between bound and unbound TF structures is considered as a key parameter in determining docking difficulty. For rigid docking in which starting TF structures are already in bound conformations, the strength of TF-DNA interaction is employed as the sole criterion for the classification.

**Table 1:** Flexible TF-DNA docking benchmark

		TF-DNA Complex							Unbound-TF				
				
		PDB			TF-DNA Unit Chains			**NRBC**^**b**^	PDB	Res.(Å)	Chains	**RMSD**_**u**_**(Å)**^**e**^	**RMSD**_**c**_**(Å)**^**e**^
										
	Name	ID	Res.(Å)	SCOP	Protein	Oligo_state	DNA						
	CopG repressor	1b01	2.56	a.43.1.3	A, B	Homodimer	E, F	5	2cpg	1.60	A, B	0.511	0.460
	PhoB	1gxp^a^	2.50	a.4.6.1	A	Monomer	C, D	7	1gxq	2.00	A	1.622	1.622
	AML1 Runt domain	1hjc	2.65	b.2.5.6	D	Monomer	E, F	6	1ean	1.70	A	1.056	1.056
	Papillomavirus E2	1jj4	2.40	d.58.8.1	A, B	Heterodimer	C, D	10	1f9f	1.90	C, D	0.949	1.484
	TATA-binding protein	1qn4	1.86	d.129.1.1	B	Monomer	E, F	15	1vok	2.10	B	0.934	0.934
	Tet repressor	1qpi	2.50	a.4.1.9	A, C	Homodimer	B, M	14	2tct	2.10	A, B	2.061	1.359
	MtaN	1r8d	2.70	a.6.1.3	A, B	Homodimer	C, D	8	1jbg	2.75	A, B	2.107	1.368
	Sigma subunit domain 4	1rio^a^	2.30	a.4.13.2	H	Monomer	U, T	6	1ku3	1.80	A	1.405	1.405
Easy	MecI	1sax	2.80	a.4.5.39	A, B	Homodimer	C, D	12	1okr	2.40	A, B	1.718	1.586
	CAP	2cgp	2.20	a.4.5.4	A, F	Homodimer	B, C, D, E	10	1i5z	1.90	A, B	1.652	1.919
	LRP/ASNC family protein	2e1c	2.10	a.4.5.32	A, F	Homodimer	B, D	12	2zny	2.59	A, B	1.339	1.184
	IdeR	2it0^a^	2.60	a.4.5.24	C, D	Homodimer	E, F	11	2isy	1.96	A, B	0.476	0.489
	Phi 434 repressor	2or1	2.50	a.35.1.2	R, L	Homodimer	A, B	17	1r69^c^	2.00	A, B	0.570	0.493
	PutA	2rbf	2.25	N/A	A, B	Homodimer	C, D	8	2gpe	1.90	A, B	0.798	0.571
	SoxR	2zhg	2.80	a.6.1.3	A, D	Homodimer	B, C	6	2zhh	3.20	A, B	1.749	1.467
	Controller protein	3clc^a^	2.80	a.35.1.3	C, D	Homodimer	E, F	14	3fya	3.00	A, B	0.834	0.809
	CprK	3e6c	1.80	a.4.5.4	C, D	Homodimer	A, B, E, F	12	3e5u	1.83	A, B	1.060	0.906
	NrtR	3gz6	2.90	N/A	A, B	Homodimer	C, D	15	3gz5	2.20	A, B	0.853	0.726
	
	Max	1an2	2.90	a.38.1.1	A, C	Homodimer	B, D	10	1r05^d^	N/A	A, B	8.074	4.767
	RXR-alpha	1by4	2.10	g.39.1.2	A, B	Homodimer	E, F	8	1rxr^c, d^	N/A	A, B	4.637	2.326
	Met repressor	1cma	2.80	a.43.1.5	A, B	Homodimer	C, D	4	1cmc	1.80	A, B	2.232	2.313
	Myb	1h8a^a^	2.23	a.4.1.3	C	Monomer	D, E	8	1gv2	1.68	A	9.153	9.153
	QacR	1jt0^a^	2.90	a.4.1.9	B, D	Homodimer	E, F	12	1jt6	2.54	D, E	2.924	1.650
	Lambda repressor	1lmb	1.80	a.35.1.2	3, 4	Homodimer	1, 2	10	1lrp	3.20	A, B	32.342	0.928
	Trp repressor	1tro^a^	1.90	a.4.12.1	A, C	Homodimer	I, J	12	1p6z	1.67	N, R	3.095	1.427
	Prospero	1xpx	2.80	a.4.1.1	A	Monomer	C, D	3	1mij	2.05	A	0.519	0.519
Hard	OhrR	1z9c	2.64	a.4.5.28	C, D	Homodimer	I, J	12	1z91	2.50	A, B	2.521	1.919
	Put3	1zme	2.50	g.38.1.1	C, D	Homodimer	A, B	5	1ajy^d^	N/A	A, B	9.326	8.725
	Phi lambda phage cII	1zs4	1.70	a.35.1.9	A, B, C, D	HT^f^	U, T	14	1zpq	2.80	A, B, C, D	4.947	2.679
	p53	2ac0	1.80	b.2.5.2	A, B, C, D	HT^f^	E, F, G, H	21	2j1y	1.69	A, B, C, D	25.325	0.932
	Omega repressor	2bnw	2.45	a.43.1.4	A, B	Homodimer	E, F	4	1irq	3.50	A, B	0.887	1.049
	ILF	2c6y	2.40	a.4.5.14	A	Monomer	C, D	8	1jxs^d^	N/A	A	2.830	2.830
	Phi 29 protein p4	2fio	2.70	N/A	A, B	Homodimer	C, D	4	2fip	2.00	C, D	0.679	0.496
	IRF-2	2irf^a^	2.20	a.4.5.23	L	Monomer	C, D	6	1irf^d^	N/A	A	3.459	3.459
	CgmR	2yvh^a^	2.50	N/A	C, D	Homodimer	E, F, G, H	10	2yve	1.40	A, B	2.663	1.599
	HipB	3dnv	2.68	N/A	B, C	Homodimer	E, T	10	2wiu	2.35	B, D	3.511	2.925
	Engrailed homeodomain	3hdd^a^	2.20	a.4.1.1	A	Monomer	C, D	4	1enh	2.10	A	0.716	0.716

^a^Has more than one binding unit

^b^NRBC: number of protein residues having side-chain contacts with DNA bases

^c^Modeled unit structure

^d^NMR structure, resolution N/A

^e^RMSD_u_: global RMSD between bound and unbound TF-units, RMSD_c_: global RMSD between bound and unbound TF-chains

^f^HT: homotetramer

**Table 2:** Rigid TF-DNA docking benchmark

		TF-DNA Complex							
		
	Name	PDB			TF-DNA Unit Chains				
						
		ID	Res. (Å)	SCOP	Protein	Oligo_state	DNA	**NRBC**^**b**^	**BSA(Å^2^)**^**c**^
	ZIF268	1aay	1.60	g.37.1.1	A	Monomer	B, C	13	960.81
	Max	1an2	2.90	a.38.1.1	A, C	Homodimer	B, D	10	933.75
	Papillomavirus E2	1jj4	2.40	d.58.8.1	A, B	Heterodimer	C, D	10	839.96
	QacR	1jt0^a^	2.90	a.4.1.9	B, D	Homodimer	E, F	12	1085.51
	Lambda repressor	1lmb	1.80	a.35.1.2	3, 4	Homodimer	1, 2	10	1105.4
	TATA-binding	1qn4	1.86	d.129.1.1	B	Monomer	E, F	15	1107.51
	Tet repressor	1qpi	2.50	a.4.1.9	A, C	Homodimer	B, M	14	973.49
	MecI	1sax	2.80	a.4.5.39	A, B	Homodimer	C, D	12	1130.16
	Trep repressor	1tro^a^	1.90	a.4.12.1	A, C	Homodimer	I, J	12	1243.06
	OhrR	1z9c	2.64	a.4.5.28	C, D	Homodimer	I, J	12	1669.81
Easy	Phi lambda phage cII	1zs4	1.70	a.39.1.9	A, B, C, D	HT^d^	U, T	14	1043.06
	p53	2ac0	1.80	b.2.5.2	A, B, C, D	HT^d^	E, F, G, H	21	1921.76
	CAP	2cgp	2.20	a.4.5.4	A, F	Homodimer	B, C, D, E	10	944.43
	LRP/ASNC family protein	2e1c	2.10	a.4.5.32	A, F	Homodimer	B, D	11	803.23
	IdeR	2it0^a^	2.60	a.4.5.24	C, D	Homodimer	E, F	11	1123.8
	Phi 434 repressor	2or1	2.50	a.35.1.2	R, L	Homodimer	A, B	17	1021.78
	CgmR	2yvh^a^	2.50	N/A	C, D	Homodimer	E, F, G, H	10	1056.55
	Controller protein	3clc^a^	2.80	a.35.1.3	C, D	Homodimer	E, F	14	1002.57
	HipB	3dnv	2.68	N/A	B, C	Homodimer	E, T	10	990.24
	CprK	3e6c	1.80	a.4.5.4	C, D	Homodimer	A, B, E, F	12	1059.42
	NrtR	3gz6	2.90	N/A	A, B	Homodimer	C, D	15	1845.4
	
	CopG repressor	1b01	2.56	a.43.1.3	A, B	Homodimer	E, F	5	573.31
	RXR-alpha	1by4	2.10	g.39.1.2	A, B	Homodimer	E, F	8	1031.94
	Met repressor	1cma	2.80	a.43.1.5	A, B	Homodimer	C, D	4	693.13
	PhoB	1gxp^a^	2.50	a.4.6.1	A	Monomer	C, D	7	739.09
	Myb	1h8a^a^	2.23	a.4.1.3	C	Monomer	D, E	8	738.59
	AML1 Runt domain	1hjc	2.65	b.2.5.6	D	Monomer	E, F	6	540.76
	MtaN	1r8d	2.70	a.6.1.3	A, B	Homodimer	C, D	8	1338.92
Hard	Sigma subunit domain 4	1rio^a^	2.30	a.4.13.2	H	Monomer	U, T	6	423.27
	Prospero	1xpx	2.80	a.4.1.1	A	Monomer	C, D	3	325.79
	Put3	1zme	2.50	g.38.1.1	C, D	Homodimer	A, B	5	1211.56
	Omega repressor	2bnw	2.45	a.43.1.4	A, B	Homodimer	E, F	4	519.26
	ILF	2c6y	2.40	a.4.5.14	A	Monomer	C, D	8	814.94
	Phi 29 protein p4	2fio	2.70	N/A	A, B	Homodimer	C, D	4	903.33
	IRF-2	2irf^a^	2.20	a.4.5.23	L	Monomer	C, D	6	668.45
	PutA	2rbf	2.25	N/A	A, B	Homodimer	C, D	8	614.12
	SoxR	2zhg	2.80	a.6.1.3	A, D	Homodimer	B, C	6	869.73
	Engrailed homeodomain	3hdd^a^	2.20	a.4.1.1	A	Monomer	C, D	4	524.73

^a^Has more than one binding unit

^b^NRBC: number of protein residues having side-chain contacts with DNA bases

^c^BSA: buried surface area in TF-DNA complexes

^d^HT: homotetramer

## Results

### Overview of benchmark test cases

There are a total of 38 test cases for our TF-DNA docking benchmarks. About 71% of the test cases have homodimer/homotetramer TF-unit structures (Tables 1 and 2). These transcription factors have less than 35% sequence identity and cover a number of different structural folds. Based on recent SCOP (Structural Classification of Proteins) annotation (release 1.75 and pre-SCOP)[44], the test cases belong to 11 different structural folds, 15 superfamilies, and 28 families (Tables 1, 2 and Additional file 2, Tables S2 and S3). At the superfamily level, the "winged-helix DNA-binding domain" and the "homeodomain-like" superfamilies are relatively overrepresented with 8 and 5 cases respectively. Even though they are in the same superfamily, different TF-DNA interaction patterns and/or degrees of structural changes between bound and unbound TF units (Additional file 1, Figure S3 and Additional file 2, Table S2) point them into different groups of docking difficulty.

### Classification of TF-DNA complexes

An ideal TF-DNA docking benchmark should have TF-DNA complexes with various degrees of difficulty. Similar to other types of docking benchmarks, the degree of conformational change represents different levels of challenge for flexible docking [29,32]. The larger structural change a TF undergoes after binding to DNA, the more difficult it is to predict the correct docking conformation due to the added complexity in conformational search space. For rigid docking test cases, this is not an issue as bound TF conformations are used (Figure 1). The conformational differences in terms of RMSD_u _between the bound and unbound TF-units of the test cases range from 0.5 Å to 32 Å (Table 1).

Another important factor that affects the accuracy of TF-DNA docking, in both the flexible and the rigid cases, is the strength of TF-DNA interaction. A weaker interaction between TF and DNA would make it more difficult for predictive programs to tell the subtle energy differences between native and wrong complex structures, leading to a high number of false positives. In this study, we examined two different metrics for the strength of TF-DNA interactions, buried surface area (BSA) and the number of residue-base contacts (NRBCs). The values of BSA range from 326 Å^2 ^to 1922 Å^2 ^while the numbers of residue-base contacts (NRBCs) go from 3 to 21 for the TF-DNA units in our benchmarks, showing the variety of TF-DNA interactions in the test cases. Since the two metrics correlate well with a Pearson's correlation coefficient of 0.73 (Additional file 1, Figure S4) and BSA includes more non-specific interaction (e.g. TF to DNA backbone interaction at TF-DNA interface) than NRBC, we use NRBC, the number of residue-base contacts, as a measure of the strength of TF-DNA interaction.

### Flexible docking benchmark

The flexible TF-DNA docking benchmark contains 37 test cases that have bound DNA conformation and unbound TF structures (Table 1). The test cases are classified into easy and hard cases based on a combination of RMSD_u _and NRBC. The criteria used for docking difficulty of the flexible docking cases are as follows (number of cases in parentheses):

Easy (18): *RMSDu ≤ 2.5Å *AND *NRBC ≥ 5*

Hard (19): *NRBC ≤ 4 *OR *RMSDu > 2.5 Å*

**Figure 3:**
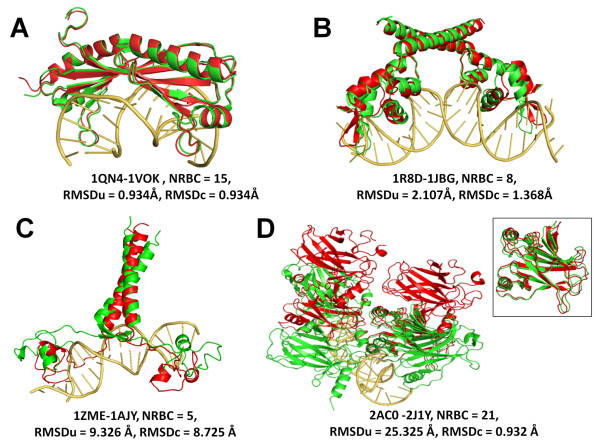
**Examples of easy and hard cases in the flexible TF-DNA docking benchmark**. A: easy case, 1QN4 (bound) -1VOK (unbound); B: easy case, 1R8D (bound)-1JBG (unbound); C: hard case, 1ZME (bound)-1AJY (unbound); D: hard case, 2AC0 (bound)-2J1Y (unbound), inset: superposition of one TF-chain from 2AC0 and one from 2J1Y. Unbound TF structures (red) were superimposed onto bound TF structures (green).

The detailed results are shown in Table 1. The easy cases have relatively strong TF-DNA interactions and small conformational changes between bound and unbound TF structures. Figure 3A shows two such examples. The TATA box binding protein, 1QN4, has 15 residue-base contacts and an RMSD_u _of 0.934 Å compared to the unbound form, 1VOK. In the case of 1R8D-1JBG pair (Figure 3B, NRBC = 8, RMSD_u _= 2.107Å), the complex has weaker TF-DNA interactions and slightly more conformational changes in transcription factors when compared to the 1QN4-1VOK pair (Figure 3A). The hard cases have fewer numbers of residue-base contacts between TF and DNA and/or large structural changes in TFs after their binding to DNA (Figures 3C and 3D). The large conformational changes in hard cases can be a result of local structural changes (RMSD_u _= 9.326Å and RMSD_c _= 8.725 Å between bound 1ZME and unbound 1AJY, Figure 3C) or the difference in the global orientation of identical TF-chain structures (RMSD_u _= 25.325 Å and RMSD_c _= 0.932 Å between bound 2AC0 and unbound 2J1Y, Figure 3D). The TF-DNA binding unit in 2AC0 (tumor-suppressor protein p53) has four identical protein chains, and the structural difference between the bound and unbound TF chains is rather small with an RMSD_c _of 0.932 Å at maximum. However, the difference between bound and unbound TF at unit-level stands over RMSD_u _of 25 Å due to the different arrangement of the identical TF chains, making it a very challenging case for flexible docking (Figure 3D).

### Rigid TF-DNA docking benchmark

There are 38 test cases in the rigid docking benchmark (Table 2). Since TF structures adopt the bound conformation in rigid docking, we only considered the interaction strength between TFs and DNA in classifying the test cases into two groups of similar sizes with different degrees of docking difficulty (number of cases in parentheses):

Easy (21): *NRBC ≥ 10*

Hard (17): *NRBC < 10*

**Figure 4:**
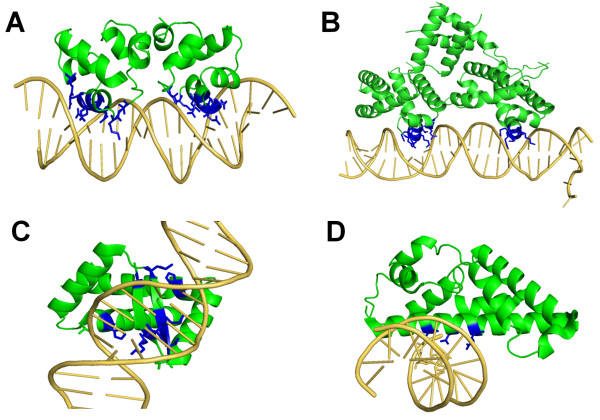
**Examples of easy and hard cases in the rigid TF-DNA docking benchmark**. A: easy case, 2OR1, NRBC = 17; B: easy case, 1ZS4, NRBC = 14; C: hard case, 2RBF, NRBC = 8; C: hard case, 1XPX, NRBC = 3. The residue side-chains that are in contact with DNA bases are rendered in blue sticks.

The benchmark has 21 easy and 17 hard cases (Table 2). Examples of easy and hard cases for rigid TF-DNA docking are shown in Figure 4. Test cases 2OR1 (repressor of phage 434) and 1ZS4 (bacteriophage lambda cII) are classified as easy cases as they have high interaction strength with NRBC of 17 and 14, respectively (Figures 4A and 4B). It is notable that 1ZS4 is considered to be a hard one in the flexible docking benchmark due to its relatively large conformational change after binding to DNA (RMSD_u _~5 Å) even though it has a large number of residue-base interactions. Multifunctional PutA (2RBF) and neural transcription factor Prospero (1XPX) are classified as hard cases, respectively, based on the small number of residue-base interactions (8 for 2RBF and 3 for 1XPX, Figures 4C and 4D).

As a proof of principal, we tested the rigid docking benchmark using our previously developed rigid-docking program PD-DOCK [13] (Additional file 2, Table S4). The prediction is considered a success if the structure with the lowest energy has an RMSD smaller than 1Å ("Conformation with the lowest energy" in Additional file 2, Table S4) when compared with the native TF-DNA complex structures. Eight easy cases were successfully predicted while only 2 hard cases were docked with better than 1Å accuracy. Interestingly three cases (1cma, 1gxp, and 1hjc) in the hard group have at least one docked conformation with an RMSD better than 1Å but with higher binding energies ("Conformation with the lowest RMSD" in Additional file 2, Table S4), highlighting the rationale of assigning degrees of difficulty in rigid docking based on the interaction strength: low interaction strength between TF and DNA is prone to high false positive docking prediction since the energy function cannot correctly discriminate the near-native conformations from wrongly docked ones. Though other docking programs implemented with different docking algorithms and energy functions may have different performance, we believe that this trend will be shared by other docking programs.

## Discussion

Transcription factors are a special group of DNA binding proteins. They are sequence-specific, yet can tolerate variations in sequence at particular sites. Though transcriptional regulation is a complicated process requiring the coordination of protein expression, protein modification, accessibility of DNA sequences, and protein-protein interaction, identification of transcription factor-binding sites on a genomic scale has been considered as a key step in understanding transcription regulatory networks and remains one of the grand challenges in post-genomic bioinformatics. Structure-based TF binding site prediction has the advantage to consider the position interdependence of TFs and the contribution of flanking sequences that are not conserved to the binding specificity [57-59]. In addition, it has been demonstrated that some transcription factors can recognize multiple distinct sequence motifs [59-61]. Therefore, a structure-based model can help us better understand the interactions between TFs and their distinct sequence motifs. To facilitate TF-DNA docking study and structure-based transcription factor binding site prediction, we present here a set of non-redundant test cases for both rigid and flexible TF-DNA docking studies. The benchmarks were designed to provide a set of diverse cases for the evaluation of TF-DNA docking methods, an essential step toward understanding the capabilities and limitations of different docking approaches.

Our benchmarks have 38 TF-DNA complexes that have less than 35% of sequence identity and spread over at least 11 SCOP structural folds. Conformational search space and scoring functions represent two key factors in predictive docking. The structural difference between bound and unbound TFs reflects the size of conformational search space for a program to explore while the interaction strength between TFs and DNA indicates how accurate and well-refined the program's scoring function should be. The common feature that we consider in assigning docking difficulty to the test cases in both the rigid and flexible TF-DNA docking benchmarks is the strength of interaction between TF and DNA. Between the two measures of TF-DNA interface area and the number of residue-base contacts, we use the number of residue-base contacts to assess the strength of more specific TF-DNA interactions, as non-specific interactions captured by TF-DNA interface area have less discriminative power for sequence-specific TF-DNA interaction. For flexible TF-DNA docking, the prediction algorithms should be able to address the conformational changes of TFs upon DNA binding, which is common to all predictive docking problems [32,62,63].

Though we only applied two key parameters for the docking difficulty assignments, several other factors have been shown to contribute to TF-DNA binding affinity and specificity. For example, besides the formation of hydrogen bonds between amino-acid sidechains and DNA bases, it has been demonstrated that DNA shape deformation or the "indirect readout" mechanism also plays important roles in protein-DNA recognition [41-43,64]. The oligomeric state of transcription factors is another important factor that can modulate the transcriptional activity. It has long been recognized that many transcription factors form homo- or hetero-oligomers to carry out their regulatory functions [65-67]. Moreover, compared to other types of DNA-binding proteins, one unique feature of transcription factors is their ability to bind degenerate DNA binding sequences [3]. The binding affinities vary between a TF and their degenerate binding sequences, suggesting different docking difficulties between a TF and these binding sites. We plan to update the test cases when more diverse TF-DNA structures become available. A new classification scheme by weighing other factors in TF-DNA docking difficulty assignment may be necessary when new bottlenecks are identified in the future.

## Conclusions

We constructed two benchmarks using a unified non-redundant set of 38 test cases for flexible and rigid TF-DNA docking respectively based on different criteria. The test cases cover diverse fold families and are classified into two groups in terms of degrees of difficulty in TF-DNA docking. We believe these benchmarks will be useful in the development of better protein-DNA interaction potentials and novel TF-DNA docking algorithms, which bears important implications to structure-based prediction of transcription factor binding sites and drug design.

## Authors' contributions

JTG conceived the project, generated the three non-redundant datasets, and wrote the manuscript. RK performed the construction and analysis of the benchmarks and was involved in the preparation of the manuscript. RIC performed the analysis of protein-DNA interactions on three datasets, RE, TF, and NS. BH performed the rigid-docking test of the benchmark. All authors read and approved the final manuscript.

## Supplementary Material

Additional file 1 **Supplementary figures for test cases**. Figure S1: an example of a TF-DNA complex structure with two binding units; Figure S2: overview of test case selection for TF-DNA docking benchmarks; Figure S3: test cases from the same superfamily but are classified in different categories; Figure S4: correlation between NRBC and the buried surface area in 38 test cases. Click here for file

Additional file 2 **supplementary tables for datasets, structural classifications, and benchmark testing**. Table S1: PDB chains for three non-redundant datasets, RE, TF, and NS; Table S2: SCOP superfamilies for the 38 test cases; Table S3: SCOP families for the 38 test cases; Table S4: docking results on the rigid-docking benchmark using PD-DOCK. Click here for file
