# Supplementary material for: Benchmarks for flexible and rigid transcription factor-DNA docking
Source: BMC Struct Biol. 2011 Nov 1;11:45. doi: 10.1186/1472-6807-11-45 (PMC3262759; doi:10.1186/1472-6807-11-45)

Figure S1. An example of a TF-DNA complex structure with two binding units.

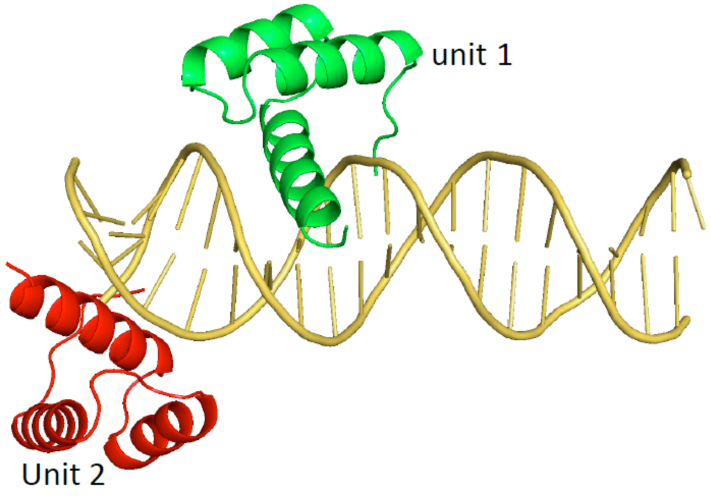

Figure S2. Overview of test case selection for TF-DNA docking benchmarks.

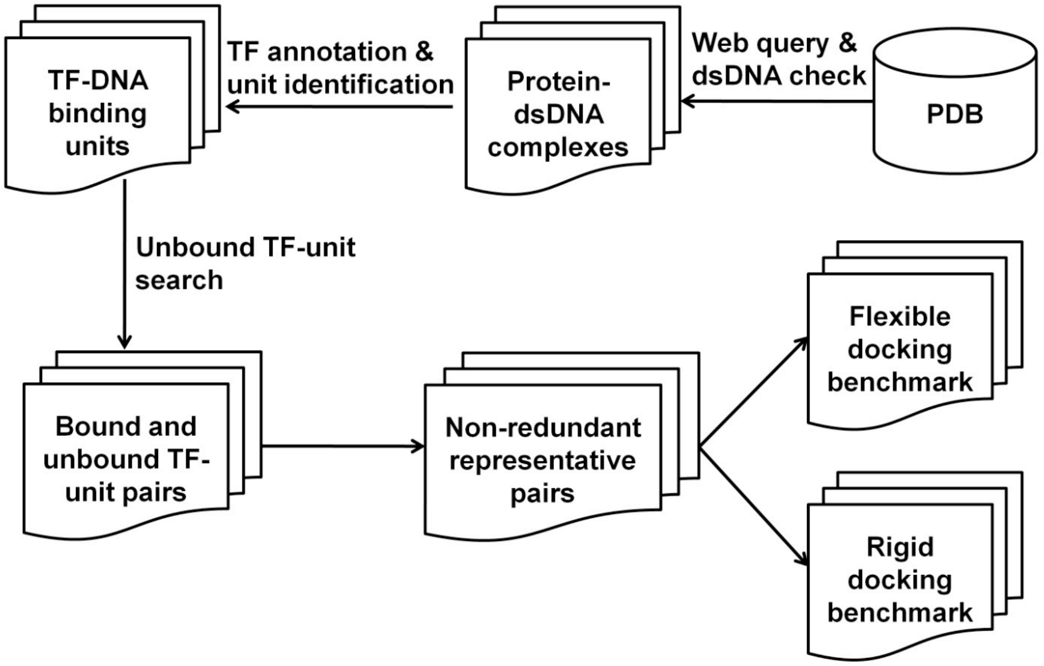

Figure S3. Test cases from the same SCOP superfamily are classified in different categories of docking difficulty. Test cases in A and B are from the same superfamily a.4.5 “Winged-helix DNA-binding domain: A(2IT0 -2ISY, RMSD=0.476 Å, NRBC=11) is an easy case while B(2IRF-1IRF, RMSD=3.459 Å, NRBC=6) is a hard case.

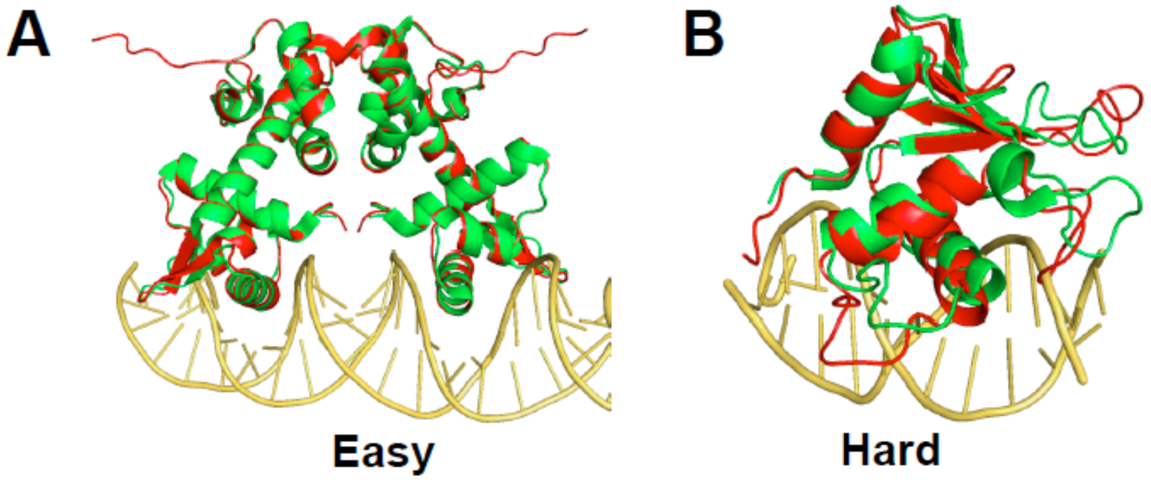

Figure S4. Correlation between NRBC and buried surface area (BSA) in 38 TF-DNA complexes.

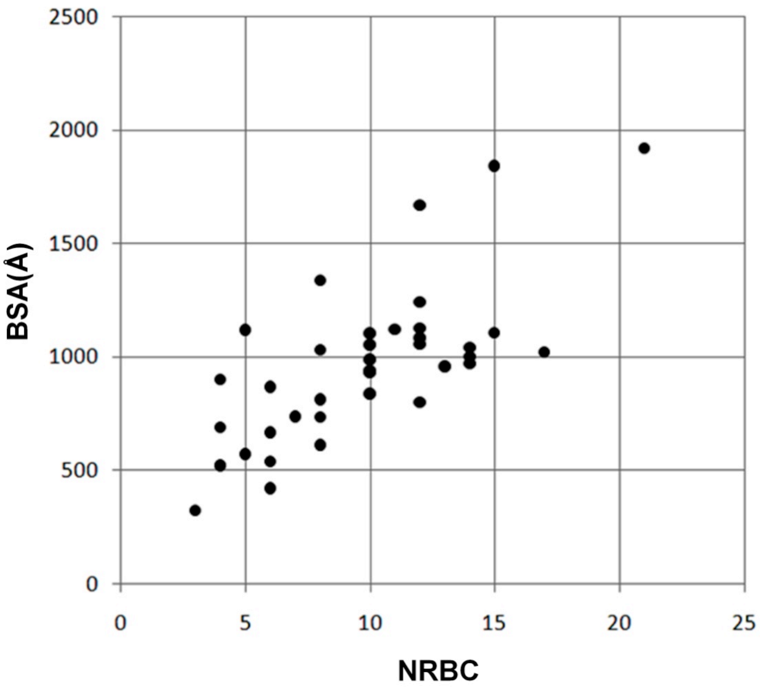

Supplement: Additional file 1 — Supplementary figures for test cases. Figure S1: an example of a TF-DNA complex structure with two binding units; Figure S2: overview of test case selection for TF-DNA docking benchmarks; Figure S3: test cases from the same superfamily but are classified in different categories; Figure S4: correlation between NRBC and the buried surface area in 38 test cases. [file 1472-6807-11-45-S1.PDF]
