# Supplementary material for: Benchmarks for flexible and rigid transcription factor-DNA docking
Source: BMC Struct Biol. 2011 Nov 1;11:45. doi: 10.1186/1472-6807-11-45 (PMC3262759; doi:10.1186/1472-6807-11-45)
Supplement: Additional file 2 — supplementary tables for datasets, structural classifications, and benchmark testing. Table S1: PDB chains for three non-redundant datasets, RE, TF, and NS; Table S2: SCOP superfamilies for the 38 test cases; Table S3: SCOP families for the 38 test cases; Table S4: docking results on the rigid-docking benchmark using PD-DOCK. [file 1472-6807-11-45-S2.PDF]

Table S1. PDB Chains of three non-redundant datasets, RE, TF, and NS

Dataset	# of chains	PDB chain IDs
RE	24	1IAWA, 1B94A, 3NDHA, 2E52A, 1SA3A, 1D2IA, 3M7KA, 2FKCA, 1D02A, 3MX4A, 1PVIA, 1BHMA, 3C25A, 1CKQA, 1FIUA, 3HQFA, 2GB7A, 1KC6A, 3OR3A, 2OAAA, 1WTEA, 1DC1A, 3BM3A, 2ODIA
TF	84	1B01A, 1RM1A, 3H0DA, 2BSQA, 2H27A, 2I13A, 1RM1C, 2C9LY, 3F27D, 3COQA, 3HTSB, 3DFXA, 2YVHA, 1EFAA, 1KU7A, 3DNVA, 1H88C, 1XPXA, 1K78A, 2RBFA, 1XSDA, 1GD2E, 1NLWA, 1JFIA, 1E3OC, 3D6YA, 1BDTA, 3ERED, 2HOSA, 1IC8A, 1SVCP, 1LQ1A, 2BSQE, 1JT0A, 1RM1B, 1JFIB, 3E6CC, 3G73A, 1TSRA, 2DGCA, 1TROA, 1AM9A, 2ETWA, 1D5YA, 1NKPA, 2E1CA, 1H6FA, 1CMAA, 1GXPA, 1U8BA, 1QPIA, 1C9BA, 3ORCA, 2BNWA, 3DNVB, 2PI0A, 3A5TA, 2NNYA, 1H0MA, 1U8RA, 2CGPA, 2R1JL, 2VY1A, 2ZHGA, 1PP7U, 1H9DA, 1RIOA, 1NGMB, 1OZJA, 1CF7B, 1BDHA, 1JE8A, 1Z9CA, 1AWCB, 1BG1A, 1ZS4A, 2FIOA, 1IO4D, 1A02N, 3GFIA, 3BS1A, 2BOPA, 2HANB, 1RZRL
NS	43	1CKTA, 2OFIA, 2O8BA, 3GQCA, 1JEYA, 3GPUA, 3JRHA, 3K4XA, 3EYIA, 2XCSB, 1KX5B, 2FMPA, 1KX5C, 2QNFA, 1EWNA, 1Z63A, 1X9MA, 3NCIA, 2Z3XA, 3MVDK, 3F2BA, 3L2PA, 1OUPA, 1KX5A, 3AAFA, 2XHBA, 1JX4A, 3M4AA, 1F4KA, 1KX5D, 3K59A, 3I0WA, 2W36A, 3KXTA, 3L4JA, 3A46A, 3MR3A, 3JXYA, 1P71A, 3CW7A, 3BEPA, 3IAYA, 2DNJA

Table S2 SCOP[a] superfamilies of the 38 test cases

SCOP identifier	SCOP superfamily name	# of test cases[b]
a.4.1	Homeodomain-like	5
a.4.5	"Winged-helix" DNA-binding domain	8
a.4.6	C-terminal effector domain of the bipartite response regulator	1
a.4.12	Trp repressor-like	1
a.4.13	Sigma3 and sigma4 domains of RNA polymerase sigma factors	1
a.6.1	Putative DNA-binding domain	2
a.35.1	Lambda repressor-like DNA-binding domains	4
a.38.1	Helix-loop-helix DNA-binding domain	1
a.43.1	Ribbon-helix-helix	3
b.2.5	p53-like transcription factors	2
d.58.8	Viral DNA-binding domain	1
d.129.1.1	TATA-box binding protein-like	1
g.37.1	Beta-beta-alpha zinc fingers	1
g.38.1	Zn2/Cys6 DNA-binding domain	1
g.39.1	Glucocorticoid receptor-like	1

[a]Based on SCOP release 1.75 and pre-SCOP.

[b]Five out of the 38 test cases do not have SCOP annotations.

Table S3. SCOP[a] families of the 38 test cases

SCOP identifier	SCOP family name	# of test cases[b]
a.4.1.1	Homeodomain	2
a.4.1.3	Myb/SANT domain	1
a.4.1.9	Tetracyclin repressor-like, N-terminal domain	2
a.4.5.4	CAP C-terminal domain-like	2
a.4.5.14	Forkhead DNA-binding domain	1
a.4.5.23	Interferon regulatory factor	1
a.4.5.24	Iron-dependent repressor protein	1
a.4.5.28	MarR-like transcriptional regulators	1
a.4.5.32	Lrp/AsnC-like transcriptional regulator N-terminal domain	1
a.4.5.39	Penicillinase repressor	1
a.4.6.1	PhoB-like	1
a.4.12.1	Trp repressor	1
a.4.13.2	Sigma4 domain	1
a.6.1.3	DNA-binding N-terminal domain of transcriptional activators	2
a.35.1.2	Phage repressors	2
a.35.1.3	SinR domain-like	1
a.35.1.9	Bacteriophage CII protein	1
a.38.1.1	Helix-loop-helix DNA-binding protein	1
a.43.1.3	CopG-like	1
a.43.1.4	Omega transcriptional repressor	1
a.43.1.5	Met repressor	1
b.2.5.2	P53 DNA-binding domain-like	1
b.2.5.6	RUNT domain	1
d.58.8.1	Viral DNA-binding domain	1
d.129.1.1	TATA-box binding protein, C-terminal domain	1
g.37.1.1	Classic zinc finger, C2H2	1
g.38.1.1	Zn2/Cys6 DNA-binding domain	1
g.39.1.2	Nuclear receptor	1

[a]Based on SCOP release 1.75 and pre-SCOP.

[b]Five out of the 38 test cases do not have SCOP annotations.

Table S4. Docking results on the rigid-docking benchmark using PD-DOCK

PDBID	Conformation with the lowest energy		Conformation with the lowest RMSD	
	$E_{Docking}$	RMSD (Å)	$E_{Docking}$	RMSD (Å)
1aay	-249.588	0.215	-248.732	0.189
1an2	-114.229	0.310	-113.855	0.289
1jj4	-142.409	0.358	-142.174	0.250
1jt0	-86.390	19.021	-62.366	12.913
1lmb	-195.738	0.175	-195.037	0.082
1qn4	-334.529	0.705	-331.829	0.288
1qpi	-86.654	16.656	-83.478	10.427
1sax	-93.411	42.342	737.125	11.154
1tro	-90.322	29.313	-83.121	15.975
1z9c	-132.768	21.390	-111.613	12.908
2cgp	-169.650	0.406	-167.957	0.281
2e1c	-63.967	5.814	-54.234	5.589
2it0	-93.817	41.948	-59.671	11.972
2or1	-255.665	0.219	-255.123	0.190
2yvh	-100.138	35.862	-80.32	19.59
3clc	-79.069	33.034	-59.208	6.099
3dnv	-154.184	0.589	-152.726	0.298
3e6c	-118.390	26.934	-83.819	11.311
3gz6	-57.063	4.098	-43.896	2.982
1b01	-8.862	19.159	3.207	5.444
1by4	-214.020	0.412	-214.020	0.412
1cma	-56.630	20.755	-51.931	0.217
1gxp	-81.984	38.925	-64.553	0.160
1h8a	-120.762	18.645	-114.818	15.030
1hjc	-65.398	25.313	-54.924	0.452
1r8d	-105.014	35.606	-76.298	16.049
1rio	-54.741	59.497	-3.032	23.878
1zme	-76.046	22.224	-61.479	15.289
2bnw	-44.264	24.153	-38.797	3.755
2c6y	-95.168	31.705	-68.356	7.551
2fio	-146.337	46.913	-46.605	18.413
2irf	-98.727	0.579	-98.030	0.450
2rbf	-52.317	36.482	-50.897	8.053
2zhg	-86.292	29.140	-57.136	11.496
3hdd	-100.741	4.555	-99.418	4.073

*The docking for 1zs4, 2ac0, and 1xpx could not be completed due to technical issues of the docking program and are omitted from the table.

The test was carried out with our previously developed protein-DNA docking program PD-DOCK [8]. The program employs a Monte-Carlo simulated annealing approach for conformational search, in which the initial temperature is set with a 0.833 acceptance rate and a cooling rate at 0.998. The search procedure incorporates (a) rotating the DNA structure in the coordinate space as a rigid body with a step size of 2 degrees and (b) translating the DNA structure in the coordinate space as a rigid body with a step size of 0.01 Å. The simulation stops when the system converges with the acceptance rate lower than 1% or the total number of steps reaches a predefined maximum (1.5 million in our current study). To increase the chance of finding the correct complex structure(s), a total of 210 independent Monte-Carlo simulations with different seeds for each protein-DNA docking were carried out.
